# Deep Learning Based Real-Time Semantic Segmentation of Cerebral Vessels and Cranial Nerves in Microvascular Decompression Scenes

**DOI:** 10.3390/cells11111830

**Published:** 2022-06-02

**Authors:** Ruifeng Bai, Xinrui Liu, Shan Jiang, Haijiang Sun

**Affiliations:** 1 Changchun Institute of Optics, Fine Mechanics and Physics, Chinese Academy of Sciences, Changchun 130033, China; bairuifeng18@mails.ucas.ac.cn (R.B.); jiangs@ciomp.ac.cn (S.J.); 2 University of Chinese Academy of Sciences, Beijing 100049, China; liuxinr@jlu.edu.cn; 3 Department of Neurosurgery, The First Hospital of Jilin University, Changchun 130021, China

**Keywords:** microvascular decompression, real-time semantic segmentation, encoder–decoder

## Abstract

Automatic extraction of cerebral vessels and cranial nerves has important clinical value in the treatment of trigeminal neuralgia (TGN) and hemifacial spasm (HFS). However, because of the great similarity between different cerebral vessels and between different cranial nerves, it is challenging to segment cerebral vessels and cranial nerves in real time on the basis of true-color microvascular decompression (MVD) images. In this paper, we propose a lightweight, fast semantic segmentation Microvascular Decompression Network (MVDNet) for MVD scenarios which achieves a good trade-off between segmentation accuracy and speed. Specifically, we designed a Light Asymmetric Bottleneck (LAB) module in the encoder to encode context features. A Feature Fusion Module (FFM) was introduced into the decoder to effectively combine high-level semantic features and underlying spatial details. The proposed network has no pretrained model, fewer parameters, and a fast inference speed. Specifically, MVDNet achieved 76.59% mIoU on the MVD test set, has 0.72 M parameters, and has a 137 FPS speed using a single GTX 2080Ti card.

## 1. Introduction

Trigeminal neuralgia (TGN) and hemifacial spasm (HFS) are the most common brain diseases. TGN is mainly manifested as short-term pain similar to an electric shock in the trigeminal nerve distribution area. A slight touch can induce TGN, seriously affecting the patient’s life quality [1]. HFS is characterized by unilateral facial muscle painless and repetitive involuntary convulsions [2], there are increasingly younger trend [3]. Microvascular decompression (MVD) is the most commonly used surgical method for the treatment of TGN [4,5] and HFS [6,7] because of its minor operative injury and obvious therapeutic effect. In 1959, Gardner and Miklo [8] first reported the successful treatment of TGN by separating the arteries that compress the trigeminal nerve during surgery. In the 1960s, Jannetta [9] reported for the first time that MVD was performed by microscope and proposed the concept of MVD. MVD is used to remove cerebral vessels from the compressed nerve by a microscopic operation and relieves the pressure of blood vessels on the nerve to achieve therapeutic purposes. When the conservative treatment effect is poor, MVD is the preferred surgical method as long as conditions permit [10]. With the progress of surgery and anesthesia technology, MVD has no clear age limit. As long as the general situation is fine, elderly patients can tolerate general anesthesia and undergo MVD treatment [11].

With the development of endoscopic technology, there is no significant difference in the efficacy of endoscopic MVD for TGN compared with MVD under a traditional microscope, but it can shorten the operation time and effectively reduce the incidence of postoperative complications [12]. Broggi et al. [13] reported that in the case of unclear neurovascular presentation under the microscope, the problem was solved by endoscopy. Endoscopy has a good light source, its wide-angle imaging can basically cover dead angles of vision, and the local amplification of endoscopy can clearly identify the responsible vessels. Endoscopy can extend the lens into the microvascular decompression area, which is not limited by the surrounding anatomical structure, and can provide a broader and clearer surgical vision image [14]. At the same time, the lens can choose multiple different angles (0°, 30°, 45°) [15]. In the operation, the lens selects the appropriate angle and the surgeons can fully explore the responsible vessels in the case of zero damage [16]. Under the condition of visualization, the location of nerve and vessel interactions can be determined and it can be ensured that the responsible vessels are sufficiently identified without stretching the brain and nerves. Then, the microscopic operation is performed to effectively reduce the incidence of tissue damage and complications. However, after successful insertion of Teflon filaments, endoscopy can be used to further observe the position between the filaments, nerves, and responsible vessels in multiple directions and without dead angles and effectively identify whether the decompression is sufficient [12] so as to accurately evaluate the surgical effect. Based on the above advantages of endoscopy, endoscopy can be used throughout MVD [17].

**Figure 1:**
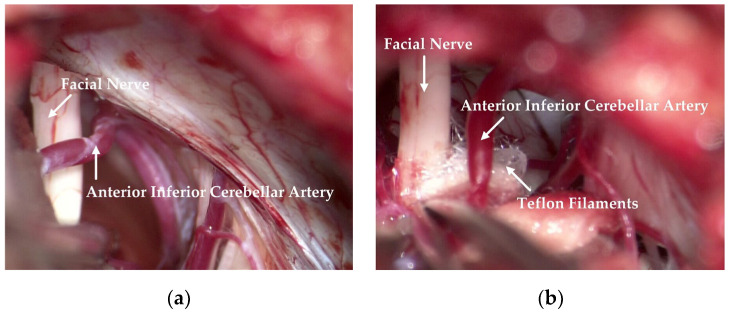
Relationship observed between the facial nerve and the responsible vessel during an operation. (**a**) The anterior inferior cerebellar artery crosses the facial nerve, resulting in compression. (**b**) Teflon filaments are inserted between the facial nerve and the anterior inferior cerebellar artery to relieve compression.

The responsible vessels are mostly the superior cerebellar artery, the anterior inferior cerebellar artery and its branches, the basilar artery, etc. Simple venous compression, arachnoid adhesion, and hypertrophy are also important factors in the pathogenesis. Attention should be paid to protecting the petrosal vein and its branches during the operation to prevent injury or tear-bleeding caused by excessive traction. The corresponding decompression method can be selected according to the thickness, elasticity, and length of the blood vessels. As shown in Figure 1, after clarifying the distribution and compression of the responsible vessels, they can then be explored with a microscope. The filler consists of Teflon filaments, which isolate the facial nerve from the responsible blood vessels so that the compression of the facial nerve is relieved. Subsequently, the filaments are observed by means of a neuro endoscope and sutured layer by layer after it has been checked that there are no actively bleeding or missing blood vessels.

Computer tomography angiography (CTA) is insufficient and there are risks of radiation, contrast-induced nephropathy, and life-threatening allergic reactions. The potential benefits of emergency CTA must be carefully weighed [18]. It should be noted that the soft-tissue resolution of CTA images is not high, which affects the diagnostic accuracy of some cerebrovascular diseases. Digital subtraction angiography (DSA) is the gold standard for the diagnosis of vascular abnormalities. However, DSA is expensive and traumatic [19] and there are various defects, such as radiation exposure, complex operation, and its time-consuming nature, which render it unsuitable for primary screening and census purposes [20]. Magnetic resonance angiography (MRA) is a non-invasive vascular imaging technique which does not require intubation or a contrast medium. With the continuous progress and development of inspection equipment and post-processing methods, the accuracy and sensitivity of detection are also increasing [19]. During and after the operation, MRA has the advantages of good soft-tissue resolution, multi-parameter imaging, multi-planar scanning, and multi-mode reconstruction. MRA can clearly show the anatomical relation between cranial nerves and responsible vessels, which is an important method for screening etiology. MRA is of great significance in the etiological diagnosis of TGN. It can accurately display the relationship between TGN and peripheral blood vessels in patients with primary trigeminal neuralgia and provide detailed anatomical details for MVD surgery. Compared with traditional CTA, DSA, MRA, and other methods used to obtain cerebral vessel images, it is simpler and more convenient to obtain true-color medical images through endoscopy, and the processing of true-color MVD images has greater development prospects.

In this paper, we propose a new real-time semantic segmentation network based on deep learning, MVDNet, which could directly identify the location of cerebral vessels and cranial nerves in MVD images quickly and accurately. MVDNet can rapidly identify cerebral vessels and cranial nerves during operations, which saves a lot of time and reduces the workloads of surgeons. Because the intra-class of cerebral vessels is too similar and the boundary between cerebral vessels and other brain tissues is not obvious, these make cerebral vessels segmentation difficult. So is the cranial nerves. These make the segmentation of cerebral vessels and nerves difficult. Therefore, we propose a more efficient encoder and a more sophisticated decoder. To train and evaluate our proposed MVDNet network, we collaborated with the First Hospital of Jilin University. We built a dataset containing 3,087 MVD images with well-annotated ground truth. MVDNet can provide real-time inferences for the segmentation and location of cerebral vessels and cranial nerves. For less experienced doctors, it helps to simplify the complexity of intraoperative operations and reduce the number of intraoperative errors. This real-time and accurate segmentation further helps doctors to obtain the best position of cerebral vessels and cranial nerves, which can assist doctors to quickly diagnose and reach the level of professional doctors even beyond professional doctors. The proposed method is compared with several other advanced methods. Experimental results show that our method has achieved the best performance.

In summary, the main contributions of this paper are as follows:

- We created a dataset consisting of 3,087 true-color MVD images and this dataset was used for the segmentation and localization of cerebral vessels and cranial nerves during MVD.
- We propose a new end-to-end network, MVDNet—a lightweight U-shaped model composed of an encoder and a decoder.
- We designed a Light Asymmetric Bottleneck (LAB) module and Feature Fusion Module (FFM) to further improve accuracy at an acceptable cost.
- Extensive experiments were conducted by comparing several of the latest methods with our proposed MVDNet and the experimental results showed that MVDNet was superior to other networks.

## 2. Related Work

**Traditional Methods:** Due to the complexity of the vascular structure, the early vessel segmentation algorithm is mainly based on intensity pattern recognition. For example, the threshold segmentation method mainly depends on the threshold value and the image is divided into different regions by a specific threshold value. Wang et al. [21] extracted foreground and background areas from MRA images through the Ostu threshold method, and divided the foreground area. This method is simple in structure and fast. It may be difficult to obtain robust and accurate segmentation results due to factors such as image noise and uneven contrast of the image. Region-growing segmentation is another commonly used vessel segmentation method. Starting from the seed points in the vessels, this method gradually adds the pixels satisfying the growth conditions in the field to a set and then realizes the vessel segmentation [22]. This method is highly dependent on the correctness of seed points and is difficult to use in automatic diagnosis systems. Some studies have used different filters for vessel enhancement, including Hessian enhancement [23], Frangi enhancement [24], Satori [25], Erdt [26], Jerman [27], matched filter [28], Gabor wavelet [29], etc. These filter methods are sensitive to noise. In addition, methods based on active contour models are also commonly used vessel segmentation methods. Cheng et al. [30] used the Snake model to complete the fundus vessel segmentation task, which improved the robustness of processing lesion data to a certain extent. Lee et al. [31] proposed a parameter active contour segmentation method based on the Kalman filter, which automatically initialized the contour curve and greatly reduced computational cost. Wang et al. [32] proposed a level set method based on adaptive thresholding by combining global and local threshold information to extract cerebral vessels from MRA data, which enhanced the extraction of small vessels.

**Deep Learning-Based Methods:** Most start-of-the-art semantic segmentation networks are based on fully convolutional neural networks (FCNs) [33]. This type of network builds a fully convolutional neural network model using convolutional layers instead of final fully connected layers, without limiting the size of the input image. The output is fused with shallow feature maps by skip connection to compensate for the detail information of the feature maps. Commonly used cerebrovascular segmentation networks include U-Net [34], ResNet [35], FCN [33], DenseNet [36], etc. Livne et al. [37] segmented the cerebral artery in TOF-MRA images by half of the number of channels in each layer of U-Net. However, this method of reducing the number of channels has limitations in detecting small blood vessels around the skull. Hilbert et al. [38] identified small vessels in TOF-MRA images by integrating 3D U-Net, multi-scale, and depth-supervised methods. This method guides the network middle layer to better generate discriminative features, avoids the problem of gradient explosion and gradient disappearance, and improves the convergence of the model. Wang et al. [39] proposed the MCANet network for the segmentation of the fetal middle cerebral artery (MCA) in ultrasound images. The network removes artifacts caused by dilated convolution through residual connection and improves the accuracy of segmentation. Wang et al. [40,41] extracted and visualized 3D cerebrovascular structures from highly sparse and noisy MRA images based on deep learning. The learned 2D multi-view slice feature vector is projected into 3D space to extract small blood vessels and improve vascular connectivity. Zhang et al. [42] introduced the reverse edge attention network to find missing cerebrovascular edge features and details, and, furthermore, improved the segmentation effect of small blood vessels. Nazir et al. [43] proposed an efficient fusion network for automatic segmentation of cerebral vessels from CTA images and used residual mapping to solve the problems of network convergence.

## 3. Method

In this section, we first introduce the light asymmetric bottleneck (LAB) module and the feature fusion module (FFM) in detail. In addition, we describe the effectiveness of these two modules. Finally, we describe the complete network architecture.

### 3.1. Light Asymmetric Bottleneck Module 

**Figure 2:**
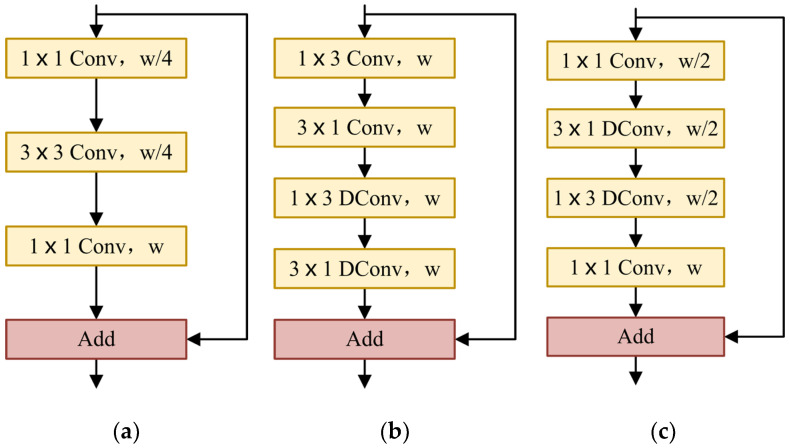
(**a**) ResNet bottleneck design. (**b**) ERFNet non-bottleneck-1D module. (**c**) Our LAB module. w: the number of input channels; DConv: depthwise dilated convolution.

In semantic segmentation tasks, some methods are implemented by maintaining the resolution of the input images to ensure sufficient spatial information [44,45,46,47]. The receptive field is also an important factor affecting segmentation. The existing methods include pyramid pooling or atrous spatial pyramid pooling to obtain large receptive fields [44,45,47,48]. Especially for real-time semantic segmentation, in order to improve the speed of the network while ensuring segmentation accuracy, it is necessary to use small input images or lightweight basic models. In this paper, we designed the light asymmetric bottleneck module (see Figure 2c) by observing the bottleneck design in ResNet [35] (see Figure 2a) and the factorized convolutions in ERFNet [49] (see Figure 2b). The LAB module has the advantages of both the bottleneck design and the factorized convolutions.

In the LAB module, we also used the bottleneck module. The LAB module is composed of 1×1 convolution, 3×1 depthwise convolution, 1×3 depthwise convolution, and the final 1×1 pointwise convolution. The residual connection is used where the number of input channels is the same as that of the output channels. The number of channels is reduced to half of the original when passing the first 1×1 convolution and the number of channels remains unchanged through 3×1 and 1×3 depthwise convolution. Finally, the original channel is restored by 1×1 pointwise convolution.

We used a 1×1 convolution at the beginning of each LAB module. For the 1×3 convolution used at the beginning of the ERFNet [49] non-bottleneck-1D module, the parameters of 1×1 convolution are far less than 1×3 convolution, which reduces the runtime and memory requirements of the network model and improves the inference speed. After the first convolution, we reduced the number of channels by half. Compared with the thousands of channels in ResNet [35], the maximum number of channels in our model is only 128, which effectively saves a lot of spatial information.

In order to further reduce the number of parameters, we referred to the non-bottleneck-1D module of ERFNet [49]. Convolutional decomposition is applied to depthwise convolution to obtain a more lightweight structure. Alvarez et al. [50] proposed that the standard convolution layer can be decomposed by 1D filters. Let $W\in {\mathbb{R}}^{C\times d^{h}\times d^{v}\times F}$ denote the weights of the typical 2D convolution layer, C be the number of input feature maps, F the number of output feature maps, and $d^{h}\times d^{v}$ the kernel size of each feature map (usually $d^{h}=d^{v}\equiv d$). Let $b\in {\mathbb{R}}^{F}$ be the vector representing the bias term for each filter. The *i*-th output of a decomposition layer $a_{i}^{1}$ can be expressed as a function of its input $a_{*}^{0}$ in the following way:

(1) $$a_{i}^{1}=\varphi \left(b_{i}^{h}+\sum_{l=1}^{L}{\bar{h}}_{il}^{T}*\left[\varphi (b_{l}^{v})+\sum_{c=1}^{C}{\bar{v}}_{lc}*a_{c}^{0}\right]\right)$$

where L denotes the number of filters in the middle layer and $\varphi \left(\cdot \right)$ is implemented by PReLU [51]. That is to say, the standard n×n depthwise convolution is replaced by n×1 depthwise convolution and 1×n depthwise convolution. For a kernel of N×N, asymmetric convolution reduces the computational complexity of each pixel from $O(N^{2})$ to O(N). At the same time, in order to extract more abundant contextual information, we introduce the dilation rates in depthwise convolution. The depthwise convolution of 3×1 and 1×3 is improved to depthwise dilated convolution, which increases the receptive field and captures more complex features without reducing the resolution of the feature maps.

Considering the shallow network models, PReLU [51] performance is slightly better than ReLU [52]. Therefore, PReLU [51] was selected as a nonlinear function in our LAB module and the pre-activation scheme was adopted [53]. Normalization is used before each nonlinear function [54]. It should be noted that the use of nonlinear layers in bottlenecks affects performance [55]. Accordingly, the nonlinear layer is eliminated after the final 1×1 pointwise convolution.

### 3.2. Feature Fusion Module

A general semantic segmentation model can be considered as a synthesis of a front-end encoder and a back-end decoder network [56]. The encoder is composed of multiple convolutional layers to obtain the overall and local features of the image. The convolutional layers and pooling layers can gradually reduce the spatial dimensions of inputted data and the feature dimensions. The decoder is composed of multiple deconvolution layers or unpooling layers and gradually restores the details and spatial dimensions of the target. The discriminable features, which are lower resolution and learned by the encoder, are semantically mapped to pixel spaces of higher resolution for pixel classification. Some decoders are simply composed of bilinear upsampling or several simple convolutions [47,57,58]. These decoders ignore low-level information, resulting in rough segmentation and low segmentation accuracy. Some decoders aggregate different stage features through complex modules and use low-level features to refine boundaries [48,59,60]. However, these decoders inevitably have the disadvantages of large calculation, high memory consumption, and slow rate.

High-level features contain semantic information. Low-level features contain rich spatial details. Due to the differences in semantic levels and spatial details, simple low-level features and high-level features are difficult to effectively integrate. Zhang et al. [58] found that introducing semantic information into low-level features and introducing spatial details into high-level features can enhance feature fusion. In this paper, we embed low-level features with rich spatial information into high-level features.

**Figure 3:**
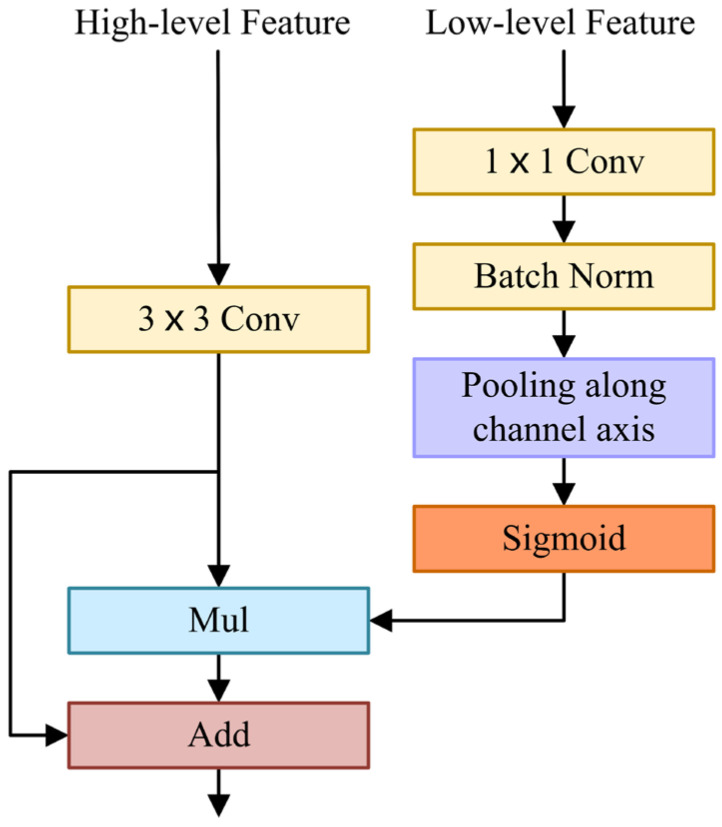
Feature Fusion Module.

The FFM uses a U-shape style to fuse low-level features with spatial information and high-level features with semantic information, as shown in Figure 3. Firstly, the low-level features are processed by 1×1 convolution and batch normalization to balance the scale of features. Then, low-level features are squeezed by an average pooling operation along the channel axis. Next, a sigmoid activation function is applied to generate a single-channel attention map. Afterwards, it is multiplied by the high-level features after 3×3 convolution. In the initial FFM, it should be noted that the high-level features are not upsampled but multiplied directly by a single-channel attention map. The subsequent two Feature Fusion Modules (FFMs) need to upsample the high-level features and multiply them with the single-channel attention map. Ultimately, the high-level features are fused with the weighted features by element addition. In short, when $F_{L}\in {\mathbb{R}}^{C_{L}\times H_{L}\times W_{L}}$ and $F_{H}\in {\mathbb{R}}^{C_{H}\times H_{H}\times W_{H}}$ are inputted as low-level and high-level features into the FFM, the final output of the FFM is computed as:

(2) $$F=\varphi \left(BN\left(\sigma \left(\left(AvgPool\left(BN\left(f^{1\times 1}\left(F_{L}\right)\right)\right)\right)\right)⊗f^{3\times 3}\left(F_{H}\right)+f^{3\times 3}\left(F_{H}\right)\right)\right)$$

where $\varphi \left(\cdot \right)$ is realized by PReLU [51], σ represents the sigmoid function, AvgPool implies the average pooling operation, BN is the batch normalization, $f^{1\times 1}$ denotes a convolution operation with the filter size of 1×1, $f^{3\times 3}$ indicates a convolution operation with a filter size of 3×3, ⊗ implies element-wise multiplication, + is element-wise addition, and $F\in {\mathbb{R}}^{C_{L}\times H_{L}\times W_{L}}$ represents the final output feature maps. 

The spatial attention map generated by low-level features reflects the importance of each pixel, which contains abundant spatial information, guides feature learning, and uses spatial details to refine boundaries. FFM extracts a spatial attention map and fuses it with high-level features containing rich semantic information effectively.

### 3.3. Network Architecture

**Figure 4:**
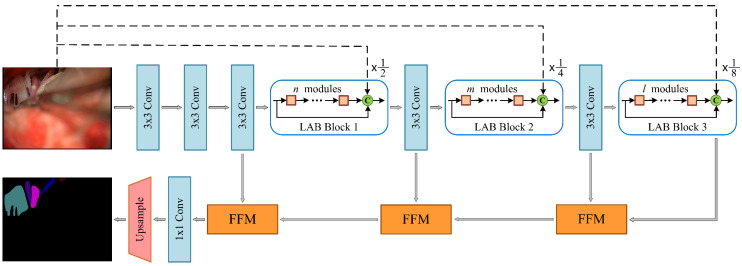
Architecture of the proposed MVDNet. C: concatenation; dashed lines indicate average pooling operations.

Based on the LAB module and FFM, we have designed the NVDNet architecture of the encoder–decoder model, as shown in Figure 4. In this section, we discuss our aim to produce a network model with fast inference speed and a high Intersection-over-Union (mIoU) metric and that is also lightweight. We analyze the optimal design of MVDNet. The detailed architecture of MVDNet is set out in Table 1.

**Encoder:** In the MVDNet, the encoder is composed of 3×3 convolution layers and LAB blocks. In the encoder, the 3×3 convolution with a step size of 2 constitutes the downsampling block. The downsampling operation reduces the size of the feature maps, expands the receptive field, and extracts more contextual information. However, it is difficult to obtain accurate segmentation results because the low resolution of feature maps leads to information loss. Therefore, in order to retain sufficient spatial information, we performed three downsampling operations on the original image to obtain 1/2, 1/4, and 1/8 feature map resolutions, respectively. Subsequently, we built a long-range shortcut connection between the input image and each LAB block, which facilitates feature reuse and compensates information loss.

We designed three LAB blocks in the MVDNet, which included several consecutive LAB modules for dense feature extraction. The first, the second, and the third LAB blocks consist of n, m, and l LAB modules, respectively. In order to strengthen the spatial relationship and feature propagation, inter-module concatenation was introduced, which realizes the fusion of high-level features and low-level features. As mentioned in Section 3.1, we applied dilated convolution in LAB modules to obtain larger receptive fields and more complex features. Finally, the input of the LAB block and the output of the LAB block are fused with the original image after downsampling, which effectively improves the feature extraction.

**Decoder:** For the decoders, we applied three feature fusion modules to aggregate low-level features and high-level features and gradually restore resolution. Next, 1×1 convolution and two-times upsampling were used to complete the segmentation. Compared to the decoder used in most semantic segmentation networks, the segmentation prediction is generally obtained by four-times [57] or eight-times [61,62] upsampling. The two-times upsampling, which we adopted, can retain more feature information as well as make the boundary information more complete and the semantic information clearer.

Our model belongs to an end-to-end deep learning architecture and does not depend on any backbone. It is noteworthy that the capacity of MVDNet is seriously low and that we use less than 0.72 million parameters.

## 4. Experiments

In this section, we evaluate our proposed network on the MVD dataset, which was provided by the First Hospital of Jilin University. Firstly, we introduce the MVD dataset and the preprocessing and implementation protocol. Then, we describe the experiments conducted on the validation set of the MVD dataset to prove the effectiveness of our network. Finally, we report the accuracy and speed results for the MVD dataset and compare them with other real-time semantic segmentation networks.

### 4.1. Data

**Figure 5:**
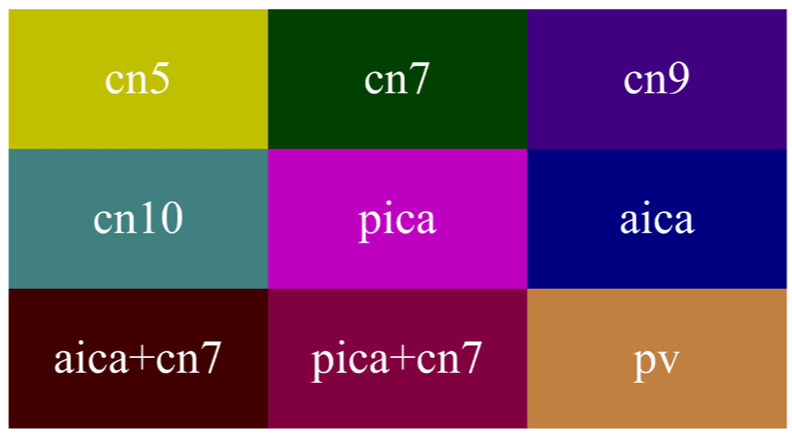
Colormap.

In the medical field, medical images generally have the characteristics of simple semantics, fewer data, and being difficult to obtain. Moreover, medical image segmentation based on deep learning requires professional annotation by doctors. Here, we cooperated with the First Hospital of Jilin University. The study involved 60 patients, 23 males and 37 females, aged 40–70. MVD data were collected with an OPMI@ VARIO 700 operation microscope produced by the manufacturer ZEISS. During the period from cerebrospinal fluid release to dura suture, 3,087 MVD images were obtained and manually labeled by experts. Then, we referred to the PASVOL VOC 2012 dataset format. Finally, the MVD dataset for semantic segmentation network training was obtained. The dataset has 9 categories (10 categories when adding background). The category names and their corresponding colors are presented in Figure 5. Abbreviations of relevant medical terms are listed in Table 2.

### 4.2. Experimental Settings

**MVD dataset:** The MVD dataset was provided by the First Hospital of Jilin University. It is an intraoperative scene dataset of microvascular decompression, including 3,087 finely annotated MVD images. We randomly selected 1,806 for training, 973 for verification, and 308 for testing. The resolution of these images was 768×576 and there were 9 categories.

**Implementation protocol:** All experiments were performed with two 2080Ti GPU cards and CUDA 10.1 and cuDNN 7.6 on the Pytorch platform. The evaluation of runtime was performed on a single 2080Ti card. Mini-batch stochastic gradient descent (SGD), with a batch size of 8, a momentum of 0.9, and a weight decay of 0.0001, was used to train the networks. We applied the “poly” learning rate policy [31], and the initial learning rate was 0.16 with power 0.9. For data augmentation, we employed random horizontal flip, random Gaussian blur, and standardization strategies. During training, we randomly cropped the input image to 512×512 and set the number of epochs to 100. The mIoU metric was used to measure accuracy. The mean of cross-entropy error over all pixels was applied as the loss.

### 4.3. Ablation Studies

In this section, we describe a series of experiments designed to prove the effectiveness of our network. These ablation studies were based on the training set of the MVD dataset and intended to evaluate our network on the validation set of the MVD dataset to observe the influence of each component in MVDNet.

**Ablation on dilation rates:** We adopted three LAB blocks with different dilation rates, LAB block 1, LAB block 2, and LAB block 3. The encoder of our network is composed of these three LAB blocks. As shown in Table 3, we set different dilation rates, 2, 4, and 8 and 4, 8, and 16. Selecting the appropriate receptive field can learn better multi-scale features. If the receptive field is too large, this will lead to the loss of small targets. For MVD images, it is more effective when the dilation rate is 2, 4, and 8.

**Ablation on downsampling of the original images:** In the encoder, we downsampled the original images by 1/2, 1/4, and 1/8 and fused them into the encoder. In Table 4, after adding the downsampling operation, the accuracy was increased from 73.49% to 73.71%. This improvement effectively preserves spatial information and details and also extracts more contextual information.

**Ablation on concatenation:** We concatenated the input features and output features of the LAB blocks. As listed in Table 4, the accuracy increased by 0.27% after adding a concatenation operation in the LAB blocks. If both downsampling and concatenation operations are introduced into the network, the accuracy reaches 73.83%. Concatenation operation is applied to the encoder, effectively increasing information flow.

**Ablation on encoder depth:** We used different numbers of LAB modules for LAB block 1, LAB block 2, and LAB block 3 to change the depth of the encoder. The paraments, FLOPs, and mIoU values of different configurations are shown in Table 5. We can see that the values of m and l have a greater impact on accuracy than n, and fine segmentation results can be obtained when m and l are superimposed with four LAB modules, respectively. When m and l increase to 8, the parameters and FLOPs increase significantly and the improvement in accuracy is minor. We made a trade-off between accuracy and computational complexity, eventually setting n to 2, m to 4, and l to 4. 

**Ablation on the decoder:** In MVDNet, we used a LAB block to extract features and chose a FFM to aggregate features. We applied the average pooling operation along the channel axis in the FFM to test the performance, as shown in Table 6. The average pooling operation along the channel axis can improve accuracy and ensure effective access to spatial details. This shows that embedding spatial details into high-level features through FFMs can effectively improve accuracy and obtain better pixel-level prediction. 

**mIoU performance:** In order to explore the influence of dilated convolution on mIoU performance, we designed two comparative experiments. In the first experiment, we removed all the dilated convolutions in MVDNet. In the other experiment, we set the first 3×3 standard convolution of the LAB block to dilated convolutions with a dilation rate of 2. As shown in Table 7, we removed all dilated convolutions and there was a significant decrease in mIoU, from 77.45% to 75.59%. When we applied dilated convolutions with a dilation rate of 2 for standard convolution, the values of mIoU also decreased (range: 77.45% to 76.76%). The experimental results show that the dilated convolution has a significant effect on mIoU performance. 

### 4.4. Comparison with the State of the Art

In this section, based on the study of ablation, we combined the LAB blocks and the FFMs to build a complete network and experimented with it on the MVD dataset. Firstly, all networks completed 100 epochs of training under the MVD dataset, the cross-entropy loss function, and mini-batch SGD (batch size: 8, momentum: 0.9, weight decay: 0.0001). Then, we conducted experiments to estimate the inference speed at a resolution of 768×576 and compared the results with those of other methods. For fair comparison, we did not adopt multi-scale or multi-crop tests.

**Figure 6:**
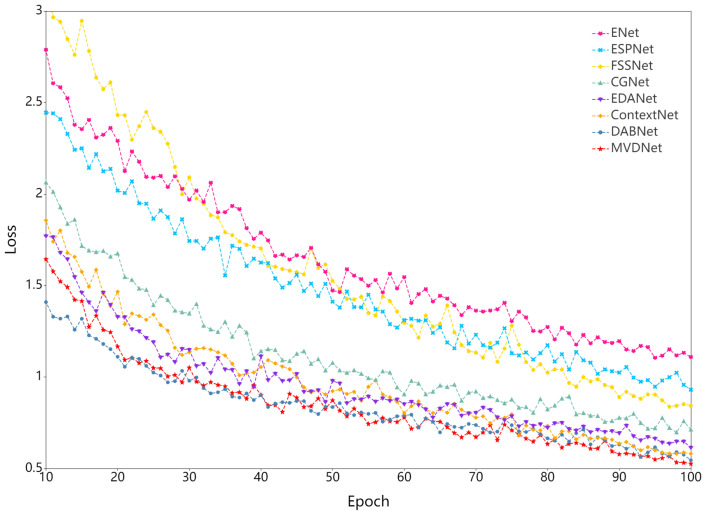
Training loss curve.

As shown in Table 8, MVDNet has 0.72 million parameters and the number of parameters is close to those of EDANet [63] and DABNet [62]. Nevertheless, the accuracy is 2.1% and 0.3% higher at the same input size. MVDNet is significantly superior to most real-time semantic segmentation methods in terms of accuracy. ESPNet [64], one of the fastest real-time networks and slightly faster than our network, only achieves 57.71% mIoU, which is 18.8% less than MVDNet. Speed comparison shows that MVDNet has a fast inference speed given the condition of ensuring accuracy. At the same time, to facilitate observation, we intercept the training loss of all networks in 10–100 epochs, as illustrated in Figure 6. From the loss curve, it can be seen that MVDNet has faster and smoother loss attenuation than the other networks after 66 epochs.

**Figure 7:**
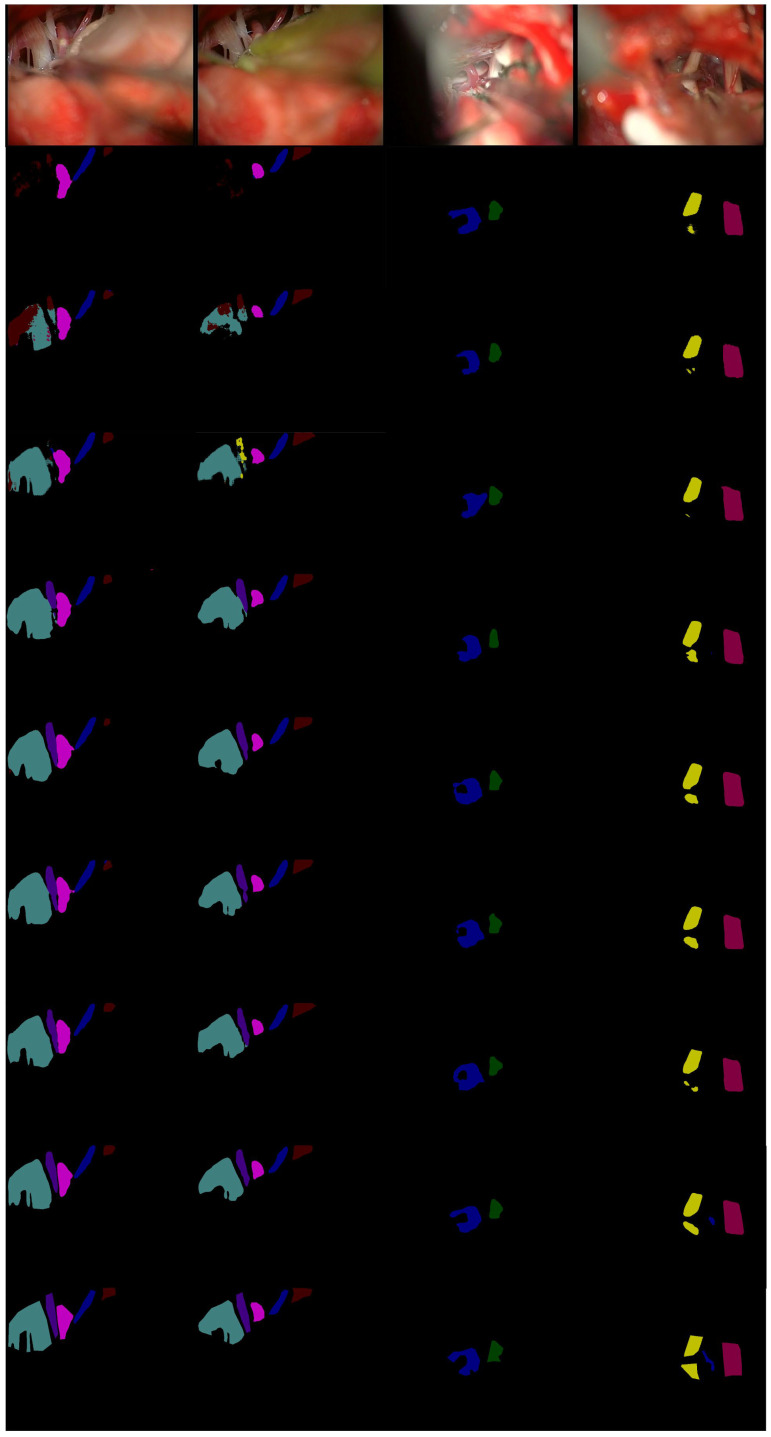
Visual comparison on MVD validation set. From top to bottom: input images, segmentation outputs from ENet [65], ESPNet [64], FSSNet [66], CGNet [67], EDANet [63], ContextNet [68], DABNet [62], our MVDNet, and ground truth.

It can be seen from Figure 7, in the first row, that only our MVDNet accurately could accurately locate the segmentation boundary of “aica” and that the object contour is clear. In the second and third rows, ENet [65] has an error segmentation and fails to segment “cn10”. ESPNet [64] and FSSNet [66] have obvious multi-pixel mixing problems. CGNet [67], EDANet [63], ContextNet [68], and DABNet [62] do not accurately locate the segmentation boundary of “cn10”, showing obvious loss of target contour segmentation. In the third row, only our MVDNet segments “aica” and the segmentation of “cn5” are complete. The proposed method has higher segmentation performance and contains more feature information.

With the test set, results were compared for patients with different background information (age and gender). According to the different age groups, there were 107 images for the 40–50-year-old group, 117 images for the 50–60 group, and 84 images for the 60–70 group. By gender, there were 124 images for males and 184 images for females.

It can be seen from Table 9 and Table 10 that the operation of MVD is mostly concentrated in “pica + cn7”, that the main responsible vessel is “pica”, and that the segmentation accuracy of cerebral vessels is less than that of cranial nerves. In Table 9, with increasing age, the segmentation accuracy of cerebral vessels is significantly reduced due to the more tortuous cerebral vessel arrangements characteristic of the elderly. In Table 10, there is a small gap between male and female mIoU values. Comparing Table 9 with Table 10, it can be found that the segmentation accuracy of cerebral vessels is mainly affected by age. Age has a greater impact on final segmentation results than gender. 

## 5. Discussion

In previous studies, there have been few real-time and accurate segmentations of cerebral vessels and cranial nerves in microvascular decompression. In this study, a new encoder–decoder structure was adopted which has the characteristics of accuracy and speed and can accurately and quickly complete the segmentation of cerebral vessels and cranial nerves in microvascular decompression. Compared with previous studies, the encoder has a simpler structure and fewer convolutional layers, yet it can obtain more contextual information. The decoder more effectively integrates high-level and low-level features.

During microvascular decompression, the surgeon usually judges the cerebral vessels and cranial nerves according to the treatment group and experience. However, the bone flap diameter is only 2.5 cm, and the operation space is small. There will also be a small amount of bleeding and cerebrospinal fluid present at any time during the operation, which will affect the operation of the surgeon and cause a lot of inconvenience, leading to many uncertainties and risks during the operation. The proposed method realizes the rapid and accurate segmentation of cerebral vessels and cranial nerves, reduces mental pressure on the surgeon, and provides a basis for rapid decision-making and judgment on the part of the surgeon. It is beneficial to reduce the release of cerebrospinal fluid during the operation, avoid excessive traction of nerves and blood vessels, effectively reduce surgical trauma, and reduce the occurrence of postoperative complications. However, the limitation of this paper is that we only used data obtained by a single medical institution (the First Hospital of Jilin University) to train, validate, and test the proposed network. The generalization performance of our approach needs to be further improved in the future.

## 6. Conclusions

For MVD images, in order to improve the speed and accuracy of real-time semantic segmentation, this paper presents the Microvascular Decompression Network (MVDNet). We have proposed a new Light Asymmetric Bottleneck (LAB) module to extract contextual information and designed an encoder based on this module. The decoder applied a Feature Fusion Module (FFM) to aggregate different features. The ablation experiments showed that the LAB blocks effectively extracted the contextual features and that the Feature Fusion Modules (FFMs) integrated the deep contextual features and shallow spatial features efficiently. We achieved a result of 76.59 % mIoU on the MVD test set at 137 FPS. Compared with other real-time methods, our network has significant improvements in terms of accuracy and speed.

## Figures and Tables

**Table 1:** Architecture details of the proposed MVDNet.

Layer	Operator	Mode	Channel	Output size
1	3×3 Conv	Stride 2	32	256×256
2	3×3 Conv	Stride 1	32	256×256
3	3×3 Conv	Stride 1	32	256×256
4–5	n×LAB module	Dilated 2	32	256×256
6	3×3 Conv	Stride 2	64	128×128
7–8	m× LAB module	Dilated 4	64	128×128
9	3×3 Conv	Stride 2	128	64×64
10–12	l× LAB module	Dilated 8	128	64×64
13	1× FFM module	-	128	64×64
14	1× FFM module	-	64	128×128
15	1× FFM module	-	32	256×256
16	1×1 Conv	Stride 1	10	256×256
17	Bilinear interpolation	×2	10	512×512

**Table 2:** Abbreviations of medical terms.

Abbreviation	Full Name in English
cn5	Trigeminal nerve
cn7	Facial nerve
cn9	Glossopharyngeal nerve
cn10	Vagus nerve
aica	Anterior inferior cerebellar artery
pica	Posterior inferior cerebellar artery
aica + cn7	Anterior inferior cerebellar artery and facial nerve
pica + cn7	Posterior inferior cerebellar artery and facial nerve
pv	Petrosal vein

**Table 3:** Results of the LAB encoder with different combinations of dilation rates.

Name	Dilation Rates	mIoU (%)
LAB_N2M2L4	2,4,8	73.49
LAB_N2M2L4	4,8,16	73.07

**Table 4:** Results of the LAB encoder with different settings. n=2, m=2, l=4.

Downsampling	Concatenation	mIoU (%)
		73.49
✓		73.71
	✓	73.76
✓	✓	73.83

**Table 5:** Results of MVDNet with different depths; the number of parameters and FLOPs are estimated for a 512×512 input.

n	m	l	Params(M)	FLOPs (G)	mIoU (%)
2	2	2	0.56	4.24	72.89
2	2	4	0.59	4.38	73.83
2	4	4	0.60	4.54	74.59
4	4	4	0.60	4.71	74.63
2	8	8	0.69	5.13	74.76

**Table 6:** Results of the FFM module with different components. n=2 , m=4, l=4.

FFM	Average Pooling	mIoU (%)
w/o	-	74.59
w		77.02
w	✓	77.45

**Table 7:** Dilation of MVDNet effect on mIoU.

Model	mIoU (%)	Params (M)
MVDNet	77.45	0.72
MVDNet_w/o dilation	75.59	0.72
MVDNet_First 3×3conv (r=2)	76.76	0.72

**Table 8:** Speed and accuracy comparison of MVDNet on the MVD test set.

Method	Params (M)	Time (ms)	Speed (fps)	mIoU (%)
ENet [65]	0.36	12.7	78.5	51.69
ESPNet [64]	0.19	6.4	156.4	57.71
FSSNet [66]	0.17	7.6	131.7	61.27
CGNet [67]	0.49	11.4	87.4	71.35
EDANet [63]	0.69	8	125	74.49
ContextNet [68]	0.88	6.1	163.3	75.62
DABNet [62]	0.75	7.7	129.1	76.29
MVDNet (Ours)	0.72	7.3	137.6	76.59

**Table 9:** Accuracy comparison for different age groups on the MVD test set.

Age	mIoU (%)	cn5	cn7	cn9	cn10	aica + cn7	pica + cn7	pica	aica	pv
40–50	76.87	82.51	82.52	74.12	76.93	77.46	88.43	74.08	71.43	64.35
50–60	77.11	82.62	84.41	79.39	81.37	73.55	87.89	73.69	70.4	60.64
60–70	76.25	84.05	87.29	75.08	81.29	79.41	88.12	70.33	68.43	52.37

**Table 10:** Accuracy comparison for gender on the MVD test set.

Gender	mIoU(%)	cn5	cn7	cn9	cn10	aica + cn7	pica + cn7	pica	aica	pv
Male	76.4	83.18	84.23	77.22	80.12	71.6	88.71	73.68	68.62	60.21
Female	76.29	85.19	85.19	73.06	74.41	78.88	87.93	72.38	71.74	60.14

## Data Availability

Restrictions apply to the availability of these data. Data were obtained from the First Hospital of Jilin University and are available from the authors with the permission of the First Hospital of Jilin University.
